# Enhanced dynamic wedge factors at off‐axis points in asymmetric fields

**DOI:** 10.1120/jacmp.v4i1.2544

**Published:** 2003-01-01

**Authors:** K. L. Prado, S. M. Kirsner, R. J. Kudchadker, R. E. Steadham, R. G. Lane

**Affiliations:** ^1^ Department of Radiation Physics The University of Texas M. D. Anderson Cancer Center 1515 Holcombe Boulevard Houston Texas 77030

**Keywords:** enhanced dynamic wedge, asymmetric field dose calculations, off‐axis dose calculations, monitor‐unit calculations

## Abstract

Several recent reports have described methods for calculating enhanced dynamic wedge factors (EDWFs). Many of these reports use the monitor‐unit (MU) fraction method to predict EDWFs as a function of field size. Although simple in approach MU fraction methods do not produce accurate EDWFs in large or asymmetric fields. A recently described technique, based on the MU fraction method works well for large and asymmetric fields, but only when the calculation point is in the center of the field. Other existing methods based on beam‐segment superposition do not have this limitation. These beam summation methods, however, are difficult to implement in routine clinical MU calculation schemes. In this paper, we present a simple calculation method that estimates EDWFs at off‐axis calculation points in both symmetric and asymmetric fields. Our method, which also is based on the MU fraction method, similarly uses empirically determined field‐size corrections but also applies wedged‐field profiles to estimate EDWFs that are independent of calculation‐point location and field symmetry. EDWF measurements for a variety of field sizes and calculation‐point locations for both 6‐ and 18‐MV x‐ray beams were performed to validate our calculations and those of our ADAC Pinnacle^3^ Treatment Planning System. The disagreement between the calculated and measured EDWFs over the useful clinical range of field sizes and calculation‐point locations was less than 2%. The worst disagreement was 3% and occurred at a point 8.5 cm from the center of an asymmetric $25(\;\text{wedged direction})\times 20{\;\text{cm}}^{2}$ 60°‐wedged field. Detailed comparisons of measurements with calculations and wedge factors obtained from the ADAC Pinnacle^3^ Treatment Planning System will be presented. In addition, the strengths and weaknesses of this calculation method will be discussed. © *2003 American College of Medical Physics.*

PACS number(s): 87.53.–j, 87.66.–a

## I. INTRODUCTION

Field wedging by dynamic means is becoming more commonplace. The introduction of the enhanced dynamic wedge (EDW) by Varian (Varian Medical Systems, Palo Alto, CA) has resulted in increased usage of dynamically wedged fields for many clinical applications. As a consequence, it is becoming desirable, and at times necessary, to be able to place calculation points and normalize treatment beams at arbitrary positions within an irradiated volume. This has made dosimetry more challenging, because these more complex monitor‐unit (MU) calculations must be independently verified. In conventionally wedged fields, this calculation has been handled using off‐axis and off‐axis‐wedge factors.^1^ The situation is somewhat more complex, however, in dynamically wedged fields, where field wedging is produced by a sweeping collimator jaw that moves during irradiation.

Jaw motion follows a pattern specified by a segmented treatment table (STT). Essentially, an STT is a table of jaw position versus cumulative monitor units. For a given wedged field, the position of the moving jaw at any point in time is a function of the selected wedge angle, the size of the field, and the transpired fraction of the total monitor‐unit setting. This process has been well described in the literature.^2^–^4^

The sweeping jaw poses a challenging dosimetry problem, because the field's size, and hence its intensity or “output factor,” changes during the irradiation. Furthermore, the field intensity varies with relative position within the field. Thus, wedge factors that are measured in the center of the symmetric fields are not easily modified so that they apply either to other positions within the symmetric field or to asymmetric field situations.

There are two general approaches to solving this problem. The first is an approximation scheme known as the MU fraction method^5^,^6^ that estimates an effective wedge factor for the particular field; the second approach is a method that considers the dynamic beam as a superposition of smaller, asymmetric beam segments.^7^,^8^ Each method has its strengths and limitations. MU fraction methods are fairly simple to implement but break down in situations where large or asymmetric fields are used or when calculation points are not in the center of the field.^9^ The beam‐segment superposition method does not have these limitations; however, this method is not easily implemented in routine MU calculation schemes.

We describe here a fairly simple method, based on the MU fraction method, that uses empirically determined field‐size corrections and applies wedged‐field profiles to estimate effective EDWFs that can be used to verify MU calculations. The proposed calculation method is validated by means of in‐phantom measurements performed at multiple calculation‐point locations within a range of symmetric and asymmetric fields. The computation method is also compared with calculations performed by a 3D treatment‐planning system.

## II. MATERIALS AND METHODS

### A. MU fraction calculation methodology

The EDW utilizes the concept of a universal wedge, wherein a wedged field of some intermediate angle *θ* is produced by the weighted sum of an open field and a 60°‐wedged field. The weighting factor used in the summation is obtained using the ratio‐of‐tangents method of Petti and Siddon:^10^

(1) $$w_{\theta }=\frac{\tan(\theta )}{\tan(60^{\circ })}.$$

In this formulation, a STT for a *θ*°‐wedged field can be computed from the Golden (60°) STT (GSTT)^2^ using the expression

(2) $$STT_{\theta }(Y)=(w_{\theta })[STT_{G}(Y)]+(w_{0})[STT_{G}(0)].$$

In the above equation, $STT_{\theta }(Y)$ is the STT value at jaw position *Y* for the *θ*° wedge, $STT_{G}(Y)$ is the GSTT value at jaw position *Y*, $STT_{G}(0)$ is the GSTT value at jaw position 0, $w_{\theta }$ is the relative 60°‐beam weight defined above, and $w_{0}$ is the relative open‐beam weight, which is given by

(3) $$w_{0}=1-w_{\theta }.$$

The jaw position, *Y*, is equal to the displacement of the moving jaw (in cm) defined at the field‐size‐definition distance (isocenter).

Our calculation method is based on the MU fraction method, which presupposes that the dynamic wedge factor at some point of reference is approximately equal to the fraction of the MU setting that the reference point is located within the open portion of the field.^3^–^6^,^9^ If $STT_{\theta }(Y)$ represents the relative MU fraction that has transpired as the jaw crosses the position *Y*, and $STT_{\theta }(Y_{f})$ is the relative MU fraction at the sweeping jaw's final position $Y_{f}$, then, according to the MU fraction method, $EDWF_{\theta }$ is approximately

(4) $$EDWF_{\theta }=\frac{STT_{\theta }(Y)}{STT_{\theta }(Y_{f})}=\frac{\left(w_{\theta })[STT_{G}(Y)]+(w_{0})[STT_{G}(0)\right]}{\left(w_{\theta })[STT_{G}(Y_{f})]+(w_{0})[STT_{G}(0)\right]}.$$

The final position, $Y_{f}$, of the sweeping jaw is located 0.5 cm from the position of the fixed jaw. Golden STTs $\left(STT_{G}\right)$ are available in tabulated form from Varian.^2^ Alternatively, analytic equations fit to these values can be used.^9^ We have chosen to do the latter.

### B. MU fraction scatter correction

Inherent in the MU fraction method is the assumption that the dose at the reference point is produced exclusively by the MUs administered before the jaw crosses the point. This assumption is not exactly correct, though the MU fraction method appears to work reasonably well for many clinical situations. In larger or asymmetric fields, and for greater wedge angles, however, EDWFs predicted using the MU fraction method differ from actual values by as much as 4%.^9^ This results from the fact that the number of MUs that are actually delivered in the first half of the sweep (before the jaw crosses the center of the field) is less than the number delivered in the second half (after the jaw crosses the center of the field). The scattered radiation contribution to the calculation point is thus underestimated, as is the resultant EDWF. This fact has been confirmed by Gibbons,^9^ who has proposed a correction scheme to overcome this shortcoming.

We, too, apply a scatter correction to EDWFs derived using the MU fraction method. However, our correction is derived from predicted‐versus‐measured EDWFs. We have measured EDWFs in the center of square and rectangular fields from $4\times 4{\;\text{cm}}^{2}$ to $20\times 20{\;\text{cm}}^{2}$ at a depth of 10 cm and have compared them with EDWFs calculated using Eq. (4) above. Ratios of actual (measured) to predicted (calculated) EDWFs were obtained for the 60° EDW for both 6 and 18 MV as a function of field length (distance from the initial moving jaw position to the fixed jaw position). A curve of the form

(5) $$Cs_{60,l}=a_{0}+(a_{1})(e^{a_{2}\times l})$$

was fit to the 60°‐EDWF ratio data using a nonlinear, least‐squares regression. In the above equation $Cs_{60,l}$ is the 60°W field‐size‐dependent scatter correction, *l* represents the field length (in the moving jaw direction), and $a_{0}$, $a_{1}$, and $a_{2}$ are coefficients determined using an exponential fit obtained using commercially available curve‐fitting software.^11^

Scatter correction factors for any other wedge angle were obtained from the 60°‐EDW scatter correction, $Cs_{60,l}$, using weighting factors obtained by the ratio of tangents method in a fashion similar to that applied to the GSTT:

(6) $$Cs_{\theta ,l}=(w_{\theta })(Cs_{60,l})+(w_{0})(Cs_{0,l}).$$

If one assumes that the scatter correction factor varies linearly with the weighting factor from 1.0 for an open (where θ=0) field, to $Cs_{60,l}$ for a 60°‐wedge field, then for a *θ*°‐wedge field,

(7) $$Cs_{\theta ,l}=w_{\theta }(Cs_{60,l}-1)+1$$

and the scatter‐corrected EDWF for a *θ*°‐wedge field becomes

(8) $$EDWF_{Cs,\theta ,l}=(EDWF_{\theta })(Cs_{\theta ,l}).$$

### C. Off‐axis corrections

The resultant scatter‐corrected EDWFs calculated thusfar apply only to calculation points that are in the center of the EDW field. Because we want to predict EDWFs at points not confined to the center of the field, we have used the wedged field's profile to model its differential position‐specific intensity. We have chosen to use the 60°‐wedge profile obtained at a depth of 10 cm for the largest square field. The profile is scaled to the field‐size definition distance (100 cm) and is normalized to 100% in the center of the field. A quadratic polynomial was least‐squares fit to the profile over the central 80% of the field using the same commercial software mentioned earlier,^11^ and an “off‐axis intensity function” of the form

(9) $$OAI_{60,x}=a_{0}+a_{1}x+a_{2}x^{2}$$

was obtained. In the above equation, $OAI_{60,x}$ is the 60°‐wedge off‐axis intensity; $a_{0}$, $a_{1}$, and $a_{2}$ are the coefficients obtained from the fit; and *x* is the distance (in cm) between the central axis of the beam and the off‐axis point.

Once again, corrections for wedge angles other than 60° were obtained by applying ratio‐of‐tangents weighting factors:

(10) $$OAI_{\theta ,x}=(w_{\theta })(OAI_{60,x})+(w_{0})(OAI_{0,x}),$$

where $OAI_{0,x}$ is the off‐axis intensity of the unwedged field at a depth of 10 cm. Since $OAI_{60,x}$ is obtained from a measured beam profile, it includes changes in the open‐field off‐axis intensity. Thus, $OAI_{0,x}$ is equal to 100%, and $OAI_{0,x}$ becomes

(11) $$OAI_{\theta ,x}=w_{\theta }(OAI_{60,x}-1)+1.$$

To calculate the EDWF at any arbitrary off‐axis calculation point located a distance $x_{p}$ from the central axis, an off‐axis wedge correction $OAWC_{\theta ,x}$ is given by

(12) $$OAWC_{\theta ,x}=\left(\frac{OAI_{\theta ,xp}}{OAI_{\theta ,xc}}\right),$$

where $x_{p}$ and $x_{c}$ are the distances from the central axis to the calculation point and to the center of the field, respectively. This correction factor is applied to the center‐of‐field EDWF.

Thus, the scatter‐corrected EDWF at a calculation point at any position relative to the central axis within a symmetric or asymmetric field is given by

(13) $$EDWF_{\theta ,l,x}=(EDWF_{\theta })(Cs_{\theta ,l})(OAWC_{\theta ,x}).$$

### D. Calculation‐method validation

The calculation method was validated by ionization‐chamber measurements made in a water phantom. A Scanditronix RFA 2000 2D scanning‐system (Scanditronix Medical AB, Uppsala, Sweden) was used. It was set at 90 cm source‐to‐surface distance (SSD) and a PTW Model 308, 0.3 cc waterproof ion chamber (Physikalisch‐Technische Werkstatatten, PTW‐New York, Hicksville, NY) was positioned at a depth of 10 cm with its long axis perpendicular to the direction of travel of the sweeping jaw. The scanning system's software was used to accurately position the chamber at all points of measurement. The chamber was connected to a CNMC Model 206 electrometer (CNMC Company, Inc., Nashville, TN), and ionization readings were integrated over accelerator settings of 100 MUs. Ionization readings were collected for both wedge orientations (by sweeping opposite jaws across the field) and were averaged. EDWFs were computed as the ratio of wedged‐field to open‐field ionization readings.

The measurements taken to validate the calculations are summarized in Table I. In all instances, the jaw defining the field width (in the unwedged direction) was fixed at 20 cm. The table shows a large, representative set of data for symmetric and asymmetric fields with a variety of center‐of‐field and off‐axis point locations. Measurements were made for the 6‐ and 18‐MV 60° EDW and for the 6‐MV 30° EDW.

### E. 3D treatment‐planning system verification

The measured EDWFs also were used to verify the ability of our ADAC Pinnacle^3^ Treatment Planning System (Version 6.0, ADAC Laboratories, Milpitas, CA) to properly model the EDW. EDWF measurements for all field sizes and at all calculation points also were compared with to EDWFs generated by the Pinnacle^3^. The water phantom data set within the Pinnacle^3^ was used, and all calculations were performed using the Collapsed Cone Convolution dose engine. The calculation points were placed at the same positions as those for the measurements. A source‐surface‐distance (SSD) of 90 cm was set to the surface of the water phantom to reproduce the measurement conditions. The prescription was set to deliver 100 MU for each calculation. Calculations were performed with open fields and then with the EDW present. The dose to each point was recorded for both conditions, and the EDWF was then derived by taking the ratio of the dose to the point with the wedge present to that without the wedge.

**Table I:** EDWF measurement conditions and calculation points. EDWF, enhanced dynamic wedge factor; CoF, center of field.

Energy (MV)	EDW angle	No. of field sizes	Symmetric/asymmetric	Center of field/off‐axis points
6	60°	12	Symmetric	12 CoF, 7 Off‐Axis
18	60°	12	Symmetric	12 CoF
6	30°	12	Symmetric	12 CoF
6	60°	6	Asymmetric	6 CoF, 20 Off‐Axis
18	60°	4	Asymmetric	4 CoF, 5 Off‐Axis
6	30°	4	Asymmetric	4 CoF, 5 Off‐Axis

## III. RESULTS

### A. Center‐of‐field data

**Figure 1:**
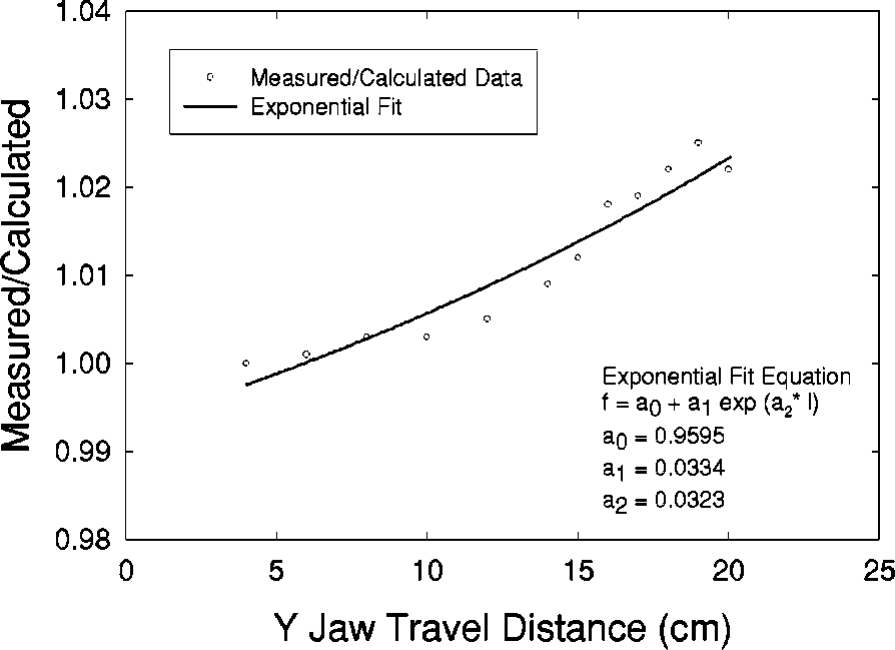
6 MV, 60° EDW scatter‐correction factor [Eq. (5)]. Shown are the data points, curve, and fit equation. The coefficient of determination $\left(r^{2}\right)$ of the regression was 0.9704.

**Figure 2:**
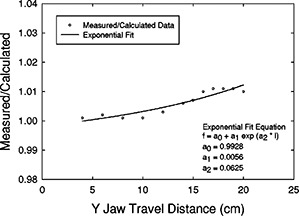
18 MV, 60° EDW scatter‐correction factor [Eq. (5)]. Shown are the data points, curve, and fit equation. The coefficient of determination $\left(r^{2}\right)$ of the regression was 0.8778.

Table II shows the comparisons of EDWFs calculated using the uncorrected MU fraction method [Eq. (4)] and measured values for both the 6‐ and 18‐MV 60° EDW at various field sizes. The ratios of measured to calculated EDWFs range from 1.0 to 1.025 for 6 MV and from 1.0 to 1.011 for 18 MV. The data are shown in detail, because these discrepancies constitute the basis for generating our scatter correction, $Cs_{60,l}$ [Eq. (6)]. The scatter correction factors used for all further calculations were obtained from a curve fit to these data. The scatter‐correction curves and equations determined for the 6‐ and 18‐MV 60° EDW are shown in Figs. 1 and 2. Scatter corrections for wedge angles other than 60° were obtained using a ratio‐of‐tangents weighting as described above.

**Table II:** Comparison of measured EDWFs with calculated, uncorrected, center‐of‐field EDWFs. Calculated EDWFs were computed using the MU‐Fraction method [Eq. (4)] without scatter corrections. Fields are symmetric.

	6‐MV, 60° EDW			18‐MV, 60° EDW		
Field size (cm^2^)	Measured	Calculated	Measured/calculated	Measured	Calculated	Measured/calculated
4×4	0.868	0.868	1.000	0.899	0.898	1.001
6×6	0.791	0.790	1.001	0.838	0.836	1.002
8×8	0.722	0.720	1.003	0.780	0.779	1.001
10×10	0.658	0.656	1.003	0.727	0.726	1.001
12×12	0.601	0.598	1.005	0.679	0.677	1.003
14×14	0.550	0.545	1.009	0.635	0.631	1.006
15×15	0.526	0.520	1.012	0.613	0.609	1.007
16×16	0.505	0.496	1.018	0.594	0.588	1.010
17×17	0.483	0.474	1.019	0.574	0.568	1.011
18×18	0.463	0.453	1.022	0.555	0.549	1.011
19×19	0.443	0.432	1.025	0.536	0.530	1.011
20×20	0.422	0.413	1.022	0.517	0.512	1.010

Table III shows an analysis of the measured‐to‐calculated EDWF ratios for points located in the center of a variety of symmetric and asymmetric fields. Calculations were performed using Eq. (8). We tested 6‐ and 18‐MV beams for both 60° and 30° EDWs. As was expected, the mean measured‐to‐calculated EDWF ratio for the 6 MV 60° EDW is 1.000 with a standard deviation of 0.003. All calculations fell within 0.5% of the measured values. Similarly, the mean measured‐to‐calculated EDWF ratio for the 18‐MV 60° EDW was 0.999 with a standard deviation of 0.004. In this case, all calculated EDWFs were within 1.3% of the measured values. Finally, the mean measured‐to‐calculated EDWF ratio for the 30° EDW for 6 MV was 1.001 with a standard deviation of 0.002. Calculated values for the 30° wedge were all within 0.4% of the measured values.

### B. Off‐axis data

**Figure 3:**
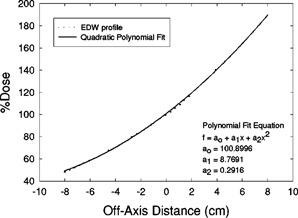
6 MV, 60° EDW off‐axis correction factor [Eq. (9)]. Shown are profile data and curve fit. Coefficients and *y*‐axis values are units of percent. The coefficient of determination $\left(r^{2}\right)$ of the regression was 0.9996.

**Figure 4:**
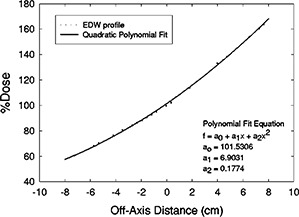
18 MV, 60° EDW off‐axis correction factor [Eq. (9)]. Shown are profile data and curve fit. Coefficients and *y*‐axis values are in units of percent. The coefficient of determination $\left(r^{2}\right)$ of the regression was 0.9993.

Figures 3 and 4 show the curves and equations that were least‐squares fit to the 10‐cm‐deep, 60° EDW profiles of the 6‐ and 18‐MV beams. These curve‐fit equations were used to apply off‐axis wedge corrections $\left(OAWC_{\theta ,x}\right)$ to the calculated EDWFs at calculation points located at

**Table III:** Comparison of measured EDWFs with calculated EDWFs after incorporation of the scatter correction $\left(Cs_{60,l}\right)$. Shown are the analyses of the ratios of center‐of‐field measured EDWFs to calculated scatter‐corrected EDWFs. EDWFs were calculated using Eq. (8). Results correspond to the center‐of‐field measurements and calculations shown in Table I.

Energy (MV)	EDW angle	Symmetric/asymmetric	Mean ratio	Standard deviation
6	60°	Symmetric	1.000	0.003
18	60°	Symmetric	1.000	0.001
6	30°	Symmetric	1.002	0.002
6	60°	Asymmetric	0.999	0.003
18	60°	Asymmetric	0.996	0.002
6	30°	Asymmetric	1.000	0.001

positions other than the geometric center of the field. Again, corrections for wedge angles other than 60° were obtained using a ratio‐of‐tangents weighting as described above.

Table IV shows the analysis of the measured‐to‐calculated EDWF ratios when calculation points are no longer restricted to the geometric center of the field. Calculations were performed using Eq. (13). These off‐axis points were located along both directions across the length of the EDW. The mean ratio of measured‐to‐calculated EDWFs for the 6‐MV 60° EDW was 1.005 with a standard deviation of 0.014. The worst case agreements, 1.029 and 1.030, occur at a point located 6 cm from the central axis of a $20\times 20{\;\text{cm}}^{2}$ field, and at a point 8.5 cm from the center of a $25\;(\text{along wedged direction})\times 20{\;\text{cm}}^{2}$ asymmetric field, respectively. The ratio data at all remaining locations were within 1.4%. The spot checks of the 18‐MV 60° EDW and the 6‐MV 30° EDW yield mean measurement‐to‐calculation ratios of 0.991 and 0.997, respectively, with all data falling within 1.2%.

Table V shows the analysis of the measured‐to‐Pinnacle^3^ calculated EDWF ratios for all points. These comparisons validated the accuracy of the ADAC Pinnacle^3^ system's EDW computation. The mean ratio of measured‐to‐Pinnacle^3^‐calculated EDWFs for the 6‐MV 60° EDW was 0.996 with a standard deviation of 0.015. Once again, the greatest discrepancy occurred at a fairly large off‐axis distance (in this case 8 cm toward the heel of the EDW for a field 25 cm long). All remaining EDWFs computed by Pinnacle^3^ were within 3% of the measured values. The mean ratio of measured‐to‐calculated EDWFs for the 18‐MV 60° EDW was 0.997 with a standard deviation of 0.007. In all these cases, the EDWFs calculated by Pinnacle3 were within 2% of the measured values. Finally, the mean ratio of measured‐to‐calculated EDWFs for the 6‐MV 30° wedge was 0.997 with a standard deviation of 0.005. In all cases, the EDWF calculated by Pinnacle^3^ was within 2%, and the majority were within 1% of the measured values.

## IV. DISCUSSION

In this work, we have extended the MU fraction methodology to include calculations of EDWFs at points not restricted to the center of the field. The accuracy of the calculation method has been suitably validated by measurements. The method applies to any deliverable EDW angle and any beam energy for which a GSTT is available. The measured data set necessary to implement the calculation methodology is relatively minimal. All that is needed is a set of measured symmetric‐field EDWFs for the 60° EDW and a single beam profile for the largest symmetric field for each energy.

Our data confirms the accuracy of ADAC Pinnacle^3^ EDW calculations, leading us to conclude that the Pinnacle^3^ EDW model represents its differential intensity quite well. Overall, the majority of Pinnacle^3^ EDWF calculations agreed with measurements to within 2% over the range of field sizes and off‐axis points that would most commonly occur clinically.

Our proposed methodology uses correction factors determined from ratios between measured data and a “pure” implementation of the MU fraction method. This, in itself, may be a possible limitation of the method. Our scatter corrections are derived from center‐of‐field data. They essentially represent the lack of scatter existing at the central axis of the field from radiation delivered during the second half of the sweep, after the jaw has crossed the field center. The magnitude of the scatter correction is, therefore, a function of the final position of the moving jaw

**Table IV:** Comparison of measured EDWFs with calculated EDWFs at off‐axis points. Both scatter corrections $\left(Cs_{\theta ,l}\right)$ and off‐axis corrections $\left(OAWC_{\theta ,x}\right)$ have been incorporated into the MU fraction calculations. Shown are the analyses of the ratios of measured EDWFs to calculated EDWFs. EDWFs were calculated using Eq. (13). Results correspond to the off‐axis measurements and calculations shown in Table I.

Energy (MV)	EDW angle	Symmetric/asymmetric	Mean ratio	Standard deviation	Max ratio	Min ratio
6	60°	Symmetric	1.012	0.010	1.029	1.003
6	60°	Asymmetric	1.001	0.012	1.030	0.986
18	60°	Asymmetric	0.991	0.003	0.997	0.988
6	30°	Asymmetric	0.997	0.002	1.000	0.995

**Table V:** Comparison of measured EDWFs with ADAC Pinnacle^3^ Treatment‐Planning System EDWFs. Shown are the analyses of the ratios of measured EDWFs to ADAC EDWFs for all irradiation conditions of Table I. CoF, center of field.

Energy (MV)	EDW angle	Symmetric/asymmetric	CoF/off‐axis points	Mean ratio	Standard deviation
6	60°	Symmetric	CoF	1.001	0.005
6	60°	Symmetric	Off‐axis	0.997	0.010
18	60°	Symmetric	CoF	1.003	0.002
6	30°	Symmetric	CoF	0.999	0.002
6	60°	Asymmetric	CoF	0.997	0.014
6	60°	Asymmetric	Off‐axis	0.993	0.021
18	60°	Asymmetric	CoF	0.990	0.005
18	60°	Asymmetric	Off‐axis	0.991	0.003
6	30°	Asymmetric	CoF	0.996	0.005
6	30°	Asymmetric	Off‐axis	0.995	0.008

as well as a function of the length of the sweep. In asymmetric fields, the field center is not located at the central axis and the STT value at that point is different than that at the central axis. Furthermore, in larger fields, the magnitude of the scatter correction is greater. Consequently, our methodology over‐estimates the correction necessary in large and fairly asymmetric fields. Our largest differences (of the order of 2–3 %) between calculations and measurements occur mostly in those situations.

Data sets were kept to the minimum that we felt was necessary to achieve a reasonable compromise between ease of commissioning and acceptable agreement between calculations and measurements over a broad range of clinical conditions. Our corrections, hence, consist of values obtained from exponential and quadratic functions that were least‐squares fit to 10‐cm depth data. At depths other than 10 cm, agreement with measurements is expected to be somewhat worse than that which is reported here. We compared shallow ($d_{\text{max}}$) and deep (20 cm) EDWF calculations to Pinnacle^3^ and found that at off‐axis points located 5 cm or greater from the center of large fields ($20\times 20{\;\text{cm}}^{2}$) differences of up to 3.5% are encountered.

Despite these limitations, agreement with measurements and with Pinnacle^3^ calculations over a variety of field sizes and calculation‐point locations has been, in general, better than 2%. At the extremes, agreement was still within 3% or so, even under the most demanding calculation conditions of large asymmetric fields and at off‐axis points located far from the center of the field. For wedge angles less than 60°, agreement improves considerably.

## V. CONCLUSION

A simple calculation method that accurately estimates the EDWF at off‐axis calculation points for both symmetric and asymmetric fields has been presented. The method is easy to implement clinically and requires a relatively minimal amount of measured data. It is an extension of the MU fraction model, in which empirically derived field‐size correction factors for scatter are incorporated, as are off‐axis corrections based on wedged‐beam profiles. The methodology has been well validated by means of comparisons with measurements and with planning‐system calculations. Overall agreement has been, on average, better than 2%.
